# Posthumous tribute to Professor Bernardo Gontijo^⋆^ (08.06.1952 – 01.06-2022)

**DOI:** 10.1016/j.abd.2022.10.003

**Published:** 2022-12-02

**Authors:** Everton Carlos Siviero do Vale, Antônio Carlos Martins Guedes

**Affiliations:** Dermatology Service, Hospital das Clínicas, Universidade Federal de Minas Gerais, Belo Horizonte, MG, Brazil

The recent loss of one of its exponents, Professor Bernardo Gontijo, has left a huge void in Brazilian dermatology, which left us after fighting a hard three-year battle against the disease that afflicted him.

The gift of teaching was awakened early in Bernardo’s life. At a very young age, while studying Medicine, he taught the subjects of the English language and biology. Already at that time, he revealed his refined teaching ability and extreme generosity in transmitting his knowledge. He graduated from medical school at Universidade Federal de Minas Gerais (UFMG), in Belo Horizonte, in 1975, having completed his medical residency in Dermatology, in 1978, at Hospital das Clínicas, Universidade de São Paulo, in São Paulo. He returned to UFMG, where he obtained a Master's Degree in Dermatology (1980) and a Ph.D. in Infectious Diseases and Tropical Medicine (1997). He worked as a professor of Dermatology in the Department of Internal Medicine of this university as of 1978, initially as a Collaborating Professor, becoming an Assistant Professor in 1981. He went through all levels of his teaching career until he became a Full Professor in 2018. He, therefore, completed 44 years of invaluable dedication to the institution.

As a physician and professor at the UFMG Hospital das Clínicas, he was responsible for the training of more than 100 dermatologists from the hospital’s medical residency program, where he worked as a preceptor in the Dermatology Service, which he coordinated for more than ten years. His main areas of expertise included medical education, clinical dermatology, pediatric dermatology, and dermatological therapy, comprehending mainly atopic dermatitis, psoriasis, hemangiomas, and cutaneous vascular malformations.

He was an advisor to many students from the Faculty of Medicine postgraduate program at UFMG and participated in examination boards for master’s and doctoral degrees in the areas of dermatological allergy, pediatric dermatology, tropical dermatology, and pigmented dermatoses, both at UFMG and at other universities in the country.

He participated intensely in hundreds of congresses, scientific meetings, and dermatology seminars in Brazil and abroad, with conferences, lectures, and presentations of clinical cases. He collaborated in the organization of numerous scientific events, having chaired the Brazilian Congress of Dermatology in 1999 and the Annual Meeting of Latin American Dermatologists in 2016. He published over 100 articles in national and foreign journals and more than 40 book chapters in Brazil and abroad.

He actively participated in professional associations and medical societies, such as the Medical Association of Minas Gerais (*Associação Médica de Minas Gerais*), the Brazilian Medical Association (*Associação Médica Brasileira*), the Minas Gerais Society of Pediatrics (*Sociedade Mineira de Pediatria*), the Brazilian Society of Pediatrics (*Sociedade Brasileira de Pediatria*) and the Brazilian Society of Dermatological Surgery (*Sociedade Brasileira de Cirurgia Dermatológica*), but especially in the Brazilian Society of Dermatology (*Sociedade Brasileira de Dermatologia*, SBD), where he was a member of the board of directors, having chaired both the national and the regional society in Minas Gerais. He was also a member of several SBD committees, especially those on Teaching and Board Certification.

Certainly, his most relevant contribution to the SBD, of which he was rightfully proud, was to the entity's scientific journal, the *Anais Brasileiros de Dermatologia*, in which he worked as a reviewer, member of the editorial board, chief editor (2004- 2008) and associate editor (2016-2020). As the chief editor, he led intense work towards the qualification of the journal, which culminated in its indexing in MEDLINE/PubMed, in 2009,^1^ and as associate editor, he made enormous efforts to improve the journal, which resulted in considerable growth of its impact factor, which reached 2.113 in 2021.^2^

Abroad, he was an active member of several dermatology societies, such as the American Dermatological Association, the American Academy of Dermatology, and the Society for Pediatric Dermatology. He had been part of the Editorial Board of the Journal of the American Academy of Dermatology since 2018.

In addition to his academic and professional activities, Bernardo enjoyed life intensely. He loved to travel, fish, and hunt, having undertaken many adventures in different regions of the state of Minas, Brazil, and abroad, throughout his trajectory. He was a person with extensive general knowledge and who valued good reading, especially authors from Minas Gerais, such as Guimarães Rosa and Pedro Nava. He also appreciated good food and, together with family and friends, he devoted himself to gastronomy, a talent he certainly inherited from his family, especially his maternal one.

Bernardo is survived by his wife Flávia and their two children, Mariana and João Renato. His grandson, Francisco, arrived shortly after his demise. Also to his family, he left a legacy of dermatology, represented by his son João Renato and his wife Flávia. A legacy he inherited from his late father, the esteemed Professor João Gontijo.

## Financial support

None declared.

## Authors’ contributions

Everton Carlos Siviero do Vale: Drafting and editing of the manuscript; critical review of the manuscript; approval of the final version of the manuscript.

Antônio Carlos Martins Guedes: Drafting and editing of the manuscript; approval of the final version of the manuscript.

## Conflicts of interest

None declared.
